# Regional cerebral cholinergic nerve terminal integrity and cardinal motor features in Parkinson’s disease

**DOI:** 10.1093/braincomms/fcab109

**Published:** 2021-05-22

**Authors:** Nicolaas I Bohnen, Prabesh Kanel, Robert A Koeppe, Carlos A Sanchez-Catasus, Kirk A Frey, Peter Scott, Gregory M Constantine, Roger L Albin, Martijn L T M Müller

**Affiliations:** 1 Department of Radiology, University of Michigan, Ann Arbor, MI 48105, USA; 2 Department of Neurology, University of Michigan, Ann Arbor, MI 48105, USA; 3 Neurology Service and GRECC, Veterans Administration Ann Arbor Healthcare System, Ann Arbor, MI 48105, USA; 4 Morris K. Udall Center of Excellence for Parkinson's Disease Research, University of Michigan, Ann Arbor, MI 48105, USA; 5 Parkinson’s Foundation Research Center of Excellence, University of Michigan, Ann Arbor, MI 48105, USA; 6 Department of Mathematics, University of Pittsburgh, Pittsburgh, PA 15260, USA; 7 Department of Statistics, University of Pittsburgh, Pittsburgh, PA 15260, USA; 8 The McGowen Institute for Regenerative Medicine, University of Pittsburgh Medical Center, Pittsburgh, PA 15260, USA; 9 Critical Path Institute, Tucson, AZ 85718, USA

**Keywords:** acetylcholine, cerebellum, medial geniculate nucleus, motor impairments, Parkinson's disease

## Abstract

Clinical effects of anti-cholinergic drugs implicate cholinergic systems alterations in the pathophysiology of some cardinal motor impairments in Parkinson’s disease. The topography of affected cholinergic systems deficits and motor domain specificity are poorly understood. Parkinson's disease patients (*n* = 108) underwent clinical and motor assessment and vesicular acetylcholine transporter [^18^F]-fluoroethoxybenzovesamicol PET imaging. Volumes-of-interest-based analyses included detailed thalamic and cerebellar parcellations. Successful PET sampling for most of the small-sized parcellations was available in 88 patients. A data-driven approach, stepwise regression using the forward selection method, was used to identify cholinergic brain regions associating with cardinal domain-specific motor ratings. Regressions with motor domain scores for model-selected regions followed by confounder analysis for effects of age of onset, duration of motor disease and levodopa equivalent dose were performed. Among 7 model-derived regions associating with postural instability and gait difficulties domain scores three retained significance in confounder variable analysis: medial geniculate nucleus (standardized β = −0.34, *t* = −3.78, *P* = 0.0003), lateral geniculate nucleus (β = −0.32, *t* = −3.4, *P* = 0.001) and entorhinal cortex (β = −0.23, *t* = −2.6, *P* = 0.011). A sub-analysis of non-episodic postural instability and gait difficulties scores demonstrated significant effects of the medial geniculate nucleus, entorhinal cortex and globus pallidus pars interna. Among 6 tremor domain model-selected regions two regions retained significance in confounder variable analysis: cerebellar vermis section of lobule VIIIb (β = −0.22, *t* = −2.4, *P* = 0.021) and the putamen (β = −0.23, *t* = −2.3, *P* = 0.024). None of the three model-selected variables for the rigidity domain survived confounder analysis. Two out of the four model-selected regions for the distal limb bradykinesia domain survived confounder analysis: globus pallidus pars externa (β = 0.36, *t* = 3.9, *P* = 0.0097) and the paracentral lobule (β = 0.26, *t* = 2.5, *P* = 0.013). Emphasizing the utility of a systems-network conception of the pathophysiology of Parkinson's disease cardinal motor features, our results are consistent with specific deficits in basal forebrain corticopetal, peduncupontine-laterodorsal tegmental complex, and medial vestibular nucleus cholinergic pathways, against the background of nigrostriatal dopaminergic deficits, contributing significantly to postural instability, gait difficulties, tremor and distal limb bradykinesia cardinal motor features of Parkinson’s disease. Our results suggest significant and distinct consequences of degeneration of cholinergic peduncupontine-laterodorsal tegmental complex afferents to both segments of the globus pallidus. Non-specific regional cholinergic nerve terminal associations with rigidity scores likely reflect more complex multifactorial signalling mechanisms with smaller contributions from cholinergic pathways.

## Teaser

Bohnen et al. investigated cholinergic PET correlates of cardinal motor symptoms in Parkinson's disease using complementary analysis approaches. Axial motor and tremor scores significantly associated with different and system-wide cholinergic pathways but evidence for distal limb bradykinesia scores was limited. No specific cholinergic transporter associations were found for rigidity scores.

## Graphical Abstract

**Graphical Abstract:**
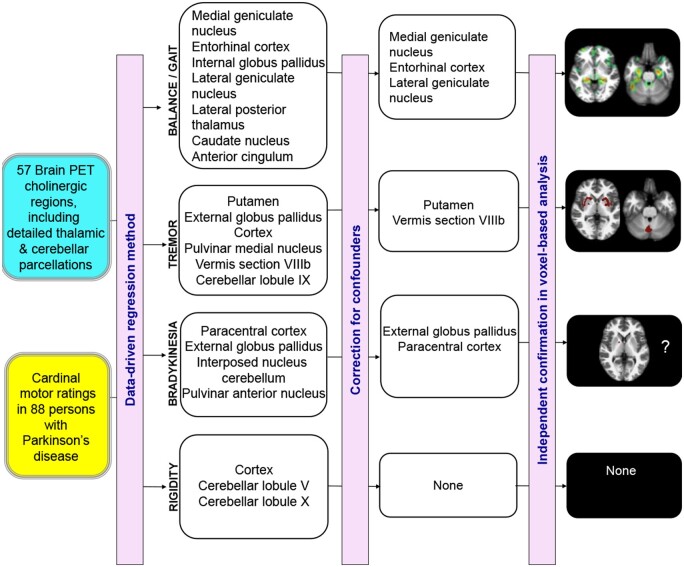

## Introduction

The cardinal motor features of Parkinson's disease are bradykinesia, tremor, rigidity and postural instability and gait difficulties (PIGD).^1^ Loss of nigrostriatal dopaminergic function is a primary pathophysiological cause of motor impairments in Parkinson's disease. Dopamine replacement therapy reliably improves bradykinesia and rigidity, but has variable impact on tremor and PIGD features, indicating that non-dopamine system deficits contribute significantly to some Parkinson's disease cardinal motor features. Although dopamine replacement therapy is the mainstay of pharmacological management of motor symptoms at the present time, anti-muscarinic cholinergic drugs were the first pharmacological agents used in the management of motor features of Parkinson's disease.^2^^,^^3^ These agents can be highly efficacious for tremor in Parkinson's disease with little benefit for bradykinesia and variable effects on rigidity.^4^ The clinical benefit of anti-muscarinic cholinergic drugs on symptoms of tremor and rigidity was explained by a model of striatal imbalance between loss of dopamine and hypothesized upregulation of cholinergic neurotransmission, at least in patients with early-stage disease.^5^^,^^6^ It is also known that these anti-cholinergic drugs may worsen PIGD motor features, including worsening freezing of gait and falls.^7–9^ These observations of beneficial and deleterious effects of anti-cholinergic drugs suggest a significant but varied role of cholinergic systems alterations in the pathophysiology of some cardinal motor impairments in Parkinson's disease.

Our recent vesicular acetylcholine transporter (VAChT) PET study found evidence of regional cholinergic terminal deficits underlying isolated episodic PIGD motor features (falls and freezing of gait) including the striatum and also the thalamus, hippocampus, amygdala and cortical regions.^10^ These observations are consistent with a framework of Parkinson's disease as a system-level disorder, with cardinal motor features emerging from deficits of and interactions between different components of the entire basal ganglia–cortex–thalamus–cerebellum system rather than from isolated basal ganglia dysfunction.^11^ A system-level model of cardinal feature pathophysiology is likely to provide a more detailed and wider topographic view of the role of acetylcholine in parkinsonian motor impairments given its ubiquitous cerebral innervation.

The objective of this study was a detailed *in vivo* examination of regional cerebral, including cortical and subcortical, VAChT [^18^F]-FEOBV (fluoroethoxybenzovesamicol) PET binding expression in Parkinson's disease subjects and correlations with ratings of the four Parkinson's disease cardinal motor features. Based on current evidence, we expect that distinct and partially overlapping subcortical (basal ganglia, thalamus, cerebellum) and cortical cholinergic terminal changes associate differentially with the four cardinal motor features of Parkinson's disease. This study expands our prior *in vivo* cholinergic imaging and clinical phenotyping studies to include not only episodic but also non-episodic PIGD motor features. We used an unbiased data-driven approach to identify key regional cholinergic terminal deficits associated with each cardinal motor feature.

## Materials and methods

### Study design and participants

This cross-sectional study involved 108 subjects with Parkinson's disease (84 males; 24 females), mean age 68.0 ± 7.6 (SD) years who were recruited from academic movement disorder clinics at the University of Michigan and the affiliated Ann Arbor VA Healthcare system during 2015–2019. Parkinson's disease subjects met the UK Parkinson’s Disease Society Brain Bank clinical diagnostic criteria.^12^ Subjects with evidence of large vessel stroke or other intracranial lesions on anatomic imaging were excluded. The movement disorder society-revised unified Parkinson's disease rating scale (MDS-UPDRS) motor examination was performed in the morning in the dopaminergic medication ‘off’ state. Subjects completed the Montreal Cognitive Assessment with mean score of 26.2 ± 3.3.^13^ Mean duration of disease was 6.0 ± 4.0 years. The mean motor examination score on the MDS-UPDRS was 35.5 ± 14.2 (range 2–74).^14^ Participants were asked about a history of falling. A fall was defined as an unexpected event during which a person falls to the ground. Thirty-five Parkinson's disease subjects were taking a combination of dopamine agonist and carbidopa-levodopa medications, 57 were using carbidopa-levodopa alone, 10 were taking dopamine agonists alone and 6 were not receiving dopaminergic drugs. No subjects were treated with anti-cholinergic or cholinesterase inhibitor drugs. Most subjects had moderate severity of disease: 6 subjects in stage 1, 3 in stage 1.5, 22 in stage 2, 43 in stage 2.5, 28 in stage 3, and 6 in stage 4 of the modified Hoehn and Yahr classification with mean stage of 2.5 ± 0.6.

PIGD motor scores were defined as the sum of MDS-UPDRS items 2.11, 2.12, 2.13, 3.10, 3.11, 3.12, 3.13 and presence (score = 1) or absence (score = 0) on the fall history question. A limited non-episodic PIGD score was also computed by omitting items related to freezing of gait and falls. Tremor scores were computed as the sum of MDS-UPDRS items 2.10, 3.15a, 3.15b, 3.16a, 3.16b, 3.17a, 3.17b, 3.17c, 3.17d, 3.17e and 3.18. Sub-scores for resting tremor and postural-kinetic tremor were also computed. MDS-UPDRS items 3.3a, 3.3b, 3.3c, 3.3d and 3.3e were summed for the rigidity score. The distal limb bradykinesia scores were based on the sum of the MDS-UPDRS items 3.4a, 3.4b, 3.5a, 3.5b, 3.6a, 3.6b, 3.7a, 3.7b, 3.8a and 3.8b.

A healthy control (HC) group consisting of 8 men and 11 women with a mean age of 67.8 ± 7.8 years was included for normative PET imaging data.

Findings of episodic mobility disturbances (i.e. freezing of gait and falls) and cognitive functions obtained in a subset of the same cohort of patients in this study have been published elsewhere.^10^^,^^15^ This study (ClinicalTrials.gov Identifier: NCT02458430 and NCT01754168) was approved by the Institutional Review Boards of the University of Michigan School of Medicine and Veterans Affairs Ann Arbor Healthcare System. Written informed consent was obtained from all subjects.

### Imaging

MRI was performed on a 3 T Philips Achieva system (Philips, Best, The Netherlands). A 3D inversion recovery-prepared turbo-field-echo was performed in the sagittal plane using TR/TE/TI = 9.8/4.6/1041 ms; turbo factor = 200; single average; FOV = 240 × 200 × 160 mm; acquired Matrix = 240 × 200 × 160 slices and reconstructed to 1 mm isotropic resolution. Magnetic resonance imaging (MRI) was performed on a 3 T Philips Achieva system (Philips, Best, The Netherlands). A 3D inversion recovery-prepared turbo-field-echo was performed in the sagittal plane using TR/TE/TI = 9.8/4.6/1041 ms; turbo factor = 200; single average; FOV = 240 × 200 × 160 mm; acquired Matrix = 240 × 200 × 160 slices and reconstructed to 1 mm isotropic resolution.

PET imaging was performed in 3D imaging mode with a Siemens ECAT Exact HR+ tomograph or Biograph 6 TruPoint PET/CT scanner (Siemens Molecular Imaging, Inc., Knoxville, TN), which acquire 63 transaxial slices (slice thickness: 2.4 mm) over a 15.2 cm axial field-of-view. Images were corrected for scatter and motion. Subjects were scanned in the dopaminergic medication ‘on’ state. [^18^F]FEOBV were prepared as described previously.^16^^,^^17^ [^18^F]-FEOBV delayed dynamic imaging was performed over 30 min (in six 5-min frames) starting 3 h after an intravenous bolus dose injection of 8 mCi [^18^F]-FEOBV.^18^

The PET imaging frames were spatially coregistered within subjects with a rigid-body transformation to reduce the effects of subject motion during the imaging session.^19^ Statistical parametric mapping (SPM) software (SPM12; Wellcome Trust Centre for Neuroimaging, University College, London, England [https://www.fil.ion.ucl.ac.uk/spm/software/spm12/]) was used for PET-MRI registration using the cropped T_1_-weighted MR volumetric scan. All brain PET images were partial volume corrected using the Müller-Gartner method^20^ and spatially normalized to Montreal Neurological Institute (MNI) template space using DARTEL normalization protocol and smoothed with a Gaussian kernel of 8 mm full width half maximum to adjust the anatomical variability between the individual brains and to enhance the signal-to-noise ratio. A supratentorial white matter reference tissue approach was used to estimate VAChT binding as previously reported.^21^^,^^22^ Distribution volume ratios were calculated from ratio of summed six delayed imaging frames (3 h after injection) for grey matter target and white matter reference tissues.^22^  Supplementary Figure 1 shows the normal cerebral biodistribution pattern of FEOBV PET.

The introduction of detailed anatomic parcellation atlases of the cerebellum and thalamic complex offers new research avenues for radioligand PET imaging studies to investigate clinical correlates of currently under-examined nuclei using this imaging modality. Freesurfer software (http://surfer.nmr.mgh.harvard.edu. Accessed June 1 2021) was used to define cortical and subcortical MR grey volumes-of-interest (VOIs) based on labels from the Mindboggle-101 dataset.^23^ Frontal, temporal, parietal and occipital cortical VOIs were computed as the average of neocortical regions with the exception of the pre-, para- and post-central cortical regions. Neocortical regions that do not uniquely belong to a specific lobe, such as the insula or fusiform gyrus, were used separately. Limbic cortical areas including the amygdala, hippocampus, entorhinal cortex, parahippocampal gyrus, anterior and posterior cingulum. Neostriatal regions included the nucleus accumbens, caudate nucleus and the putamen. We used edge erosion and only used the surviving voxels for VOI binding assessment. VOIs with a minimum acceptance criterion of at least five surviving voxels (3.8 mm^3^ per voxel) were included in our study to overcome the partial volume effect due to possible spillover from adjacent regions. We calculated VOI distribution volume ratio by averaging the distribution volume ratio values of each voxel in that region.

A limitation of the Mindboggle-101 dataset is the lack of detailed parcellation of the cerebellum and thalamic complex, two critical structures of the proposed system-level Parkinson's disease model^11^ that also show heterogeneous VAChT biodistribution patterns.^24^ For this reason, we used the SUIT toolbox (version 3.2) for cerebellar segmentation.^25^ A total of 20 cerebellar atlas regions that survived our five voxel threshold were included in the analysis: bilaterally averaged hemispheric lobules I–IV, V, VI, VIIa crus I, VIIa crus II, VII b, VIIIa, VIIIb, IX and X with vermis sections of VI, VIIa crus I, VIIa crus II, VII b, VIIIa, VIIIb, IX and X and the deep cerebellar nuclei. PET sampling of the parcellated VOIs meeting the minimum acceptance criterion was achieved in most patients (>80%) except the vermis section of crus I, which was excluded from the analysis. We used a novel probabilistic atlas of 26 thalamic nuclei built using *ex vivo* brain MRI scans and histological data.^26^ Thalamic parcellation of the PET images did not meet minimum acceptance criteria in the paracentral, paratenial, reticular and ventromedial nuclei in all patients due to small size. The following nuclei also failed in the majority of patients in the central lateral, laterodorsal, limitans and ventral anterior magnocellular nucleus. Therefore, these nuclei were excluded from the analysis leaving a total of 17 bilaterally averaged thalamic VOIs available in most patients (>80%): anteroventral nucleus, central medial nucleus, centromedian nucleus, lateral geniculate nucleus (LGN), lateral posterior nucleus, mediodorsal lateral parvocellular nucleus, mediodorsal medial magnocellular nucleus, medial geniculate nucleus (MGN), parafascicular nucleus, pulvinar anterior nucleus, pulvinar inferior nucleus, pulvinar lateral nucleus, pulvinar medial nucleus, ventral anterior nucleus, ventral lateral anterior nucleus, ventral lateral posterior nucleus and ventral posterolateral nucleus.

We delineated the GPi from the pars externa (GPe) by obtaining a mask from the standard globus pallidus probabilistic basal ganglia atlas^27^ and by transforming into individual subject space. These masks were used to selectively sample radiotracer binding for each pars within the Freesurfer globus pallidus VOI. Edge erosion was used to avoid scattered radiotracer activity from high binding putaminal regions. VOI analysis was performed in the subject's native space. The total number of VOIs used for analysis was 57 (see Supplementary Table 1 of listing of all VOIs and the respective volumes).

### Statistical analysis

Our primary analysis was VOI-based. We used a data-driven approach to select key cholinergic brain regions associating with each cardinal motor domain. Given the large number of VOI variables stepwise multiple regression using forward selection was used to select key VAChT PET VOI variables (model entry and retention criterion of *P* = 0.05 each) associating with motor ratings of specific cardinal motor features. Shapiro–Wilk test of normality was performed to confirm normality of the distribution of the residuals of the four cardinal motor variables. Non-collinearity was confirmed by a variance of inflation factor <5. The model-generated list of selected VOI regions were then used for confounder analysis using LED (levodopa equivalent dose), age of onset and duration of motor disease as covariates. Statistical analyses were performed in SAS version 9.4, SAS institute, Cary, NC. For domain-specific VOI variables that survived confounder analysis supplementary whole-brain voxel-based [^18^F]FEOBV PET analyses were performed using SPM12 software.

### Data availability

The data that support the findings of this study are available from the corresponding author, upon reasonable request.

## Results

### Clinical and demographic data and data-driven approach

**Figure 1:**
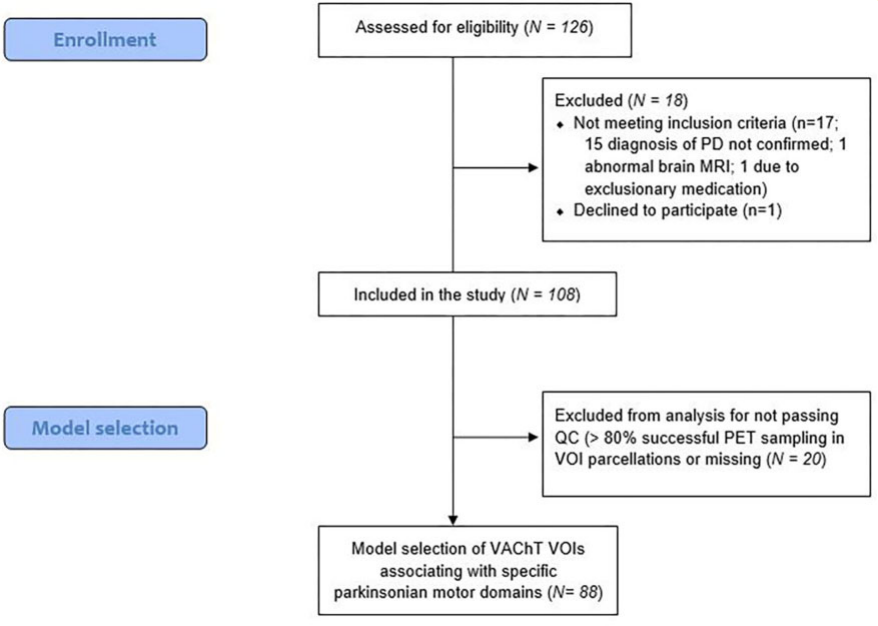
**Flowchart of the study**. Flow diagram describing the number of participants who were enrolled in the study, who were excluded or dropped out of the study, or excluded from further analysis.

A total of 108 Parkinson's disease subjects were included in the study cohort. The number of subjects with incomplete PET VOI sampling data was 20, leaving 88 subjects available for analysis (Fig. 1). Demographic and clinical variables of these 88 subjects were similar to the original study population (Fig. 1; Table 1).

**Table 1:** Summary of the demographic and clinical information of the study participants in the different subsets.

	Total patient population (*n* = 108)	Subset with minimum set of VOI anatomic parcellations (*n* = 88)
Age (years)	68.0 ± 7.6	68.0 ± 7.6
Gender (M/F)	84/24	69/19
Duration of disease (years)	6.0 ± 4.0	6.0 ± 3.8
Hoehn and Yahr stage	2.5 ± 0.6	2.6 ± 0.6
MDS-UPDRS total motor scores	35.5 ± 14.2	36.5 ± 14.2
MDS-UPDRS total PIGD motor scores	6.5 ± 4.5	6.9 ± 4.5
MDS-UPDRS total tremor scores	8.0 ± 5.3	8.1 ± 5.4
MDS-UPDRS total rigidity scores	6.7 ± 3.1	6.8 ± 3.1
MDS-UPDRS total distal limb bradykinesia scores	14.1 ± 6.9	14.5 ± 7.1
Montreal Cognitive Assessment score	26.2 ± 3.3	26.1 ± 3.4
Levodopa equivalent dose (mg)	660 ± 413	683 ± 430

Findings show that the different subsets of participants were comparable to each other. Movement Disorder Society; MDS-UPDRS, Movement Disorder Society Revised Unified Parkinson's Disease Rating Scale. Means and standard deviations are presented for numerical variables.

**Figure 2:**
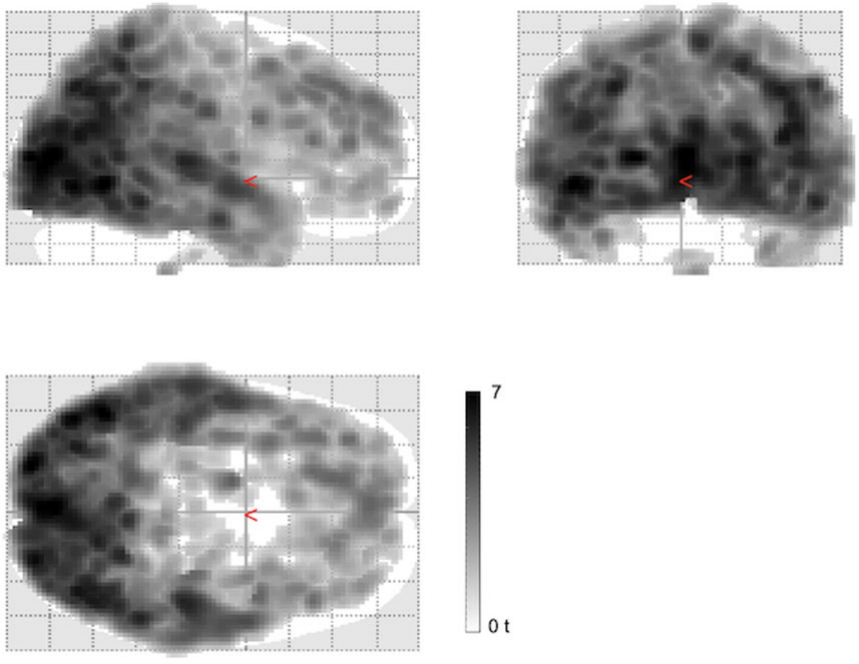
**FEOBV PET binding differences between the Parkinson's disease versus the normal control group**. The glass brain images show evidence of widespread cortical (especially in the posterior cortices), and subcortical (especially thalamic and pallidal regions) but not in striatal or limbic regions. Significant findings after false discovery rate correction (*P* < 0.05) are shown. Maximum *t*-score difference between two groups is 7.

A group comparison between the Parkinson’s disease subjects and normal control persons show widespread cortical (especially in the posterior cortices), and subcortical (especially thalamic and pallidal regions) but not in striatal or limbic regions in the patients. Significant findings after false discovery rate correction (*P* < 0.05) are shown. Maximum *t*-score difference between two groups is 7 (Fig. 2).

Table 2 shows results of the data-driven approach to select regional cholinergic terminal densities associated with each cardinal motor feature.

**Table 2:** Results of a data-driven selection approach

	Partial	Model		
Selected VAChT VOI	*R*-Square	*R*-Square	*F*-value	*P*-value
Total PIGD ratings (non-episodic and episodic)				
Medial geniculate nucleus	0.3239	0.3239	41.21	<0.0001
Entorhinal cortex	0.0596	0.3836	8.22	0.0052
Globus pallidus pars interna	0.0353	0.4189	5.1	0.0265
Lateral geniculate nucleus	0.0302	0.4491	4.55	0.036
Lateral posterior nucleus thalamus	0.0391	0.4881	6.26	0.0144
Caudate nucleus	0.0316	0.5198	5.34	0.0234
Anterior cingulum	0.0251	0.5449	4.41	0.0388
Tremor ratings				
Putamen	0.084	0.084	7.89	0.0062
Globus pallidus pars externa	0.0651	0.1491	6.5	0.0126
(Occipital) cortex	0.0562	0.2053	5.94	0.0169
Pulvinar medial nucleus	0.0571	0.2624	6.42	0.0131
Cerebellar vermis section of lobule VIIIb	0.0502	0.3126	5.99	0.0165
Cerebellar hemispheric section of lobule IX	0.0326	0.3452	4.03	0.0479
Rigidity ratings				
(Occipital) cortex	0.0875	0.0875	8.25	0.0051
Cerebellar hemispheric section of lobule V	0.0451	0.1327	4.42	0.0384
Cerebellar lobule X	0.0643	0.197	6.73	0.0112
Distal limb bradykinesia ratings				
Paracentral cortex	0.1116	0.1116	10.8	0.0015
Globus pallidus pars externa	0.0764	0.188	8	0.0058
Interposed nucleus (cerebellum)	0.0742	0.2622	8.45	0.0047
Pulvinar anterior nucleus	0.041	0.3032	4.89	0.0298

Results of the data-driven selection approach shows cholinergic correlates of cardinal motor domains in Parkinson's disease. Bilaterally averaged VOI regions are presented except for the (midline) cerebellar vermis.

### PET-clinical correlations of the model-selected variables and *post hoc* analysis: PIGD motor domain

Multiple regression with forward selection identified 7 VOIs where cholinergic terminal changes correlated with PIGD ratings (Table 2). *Post hoc* analysis was performed to assess for possible confounder effects of age of onset, duration of disease and LED in multiple regression models. Analysis for the MGN independent variable showed a significant model [*F*(4,98) = 12.1, *P* < 0.0001] with significant effects for MGN VAChT expression (standardized β = −0.34, *t* = −3.78, *P* = 0.0003) and significant covariate effects for duration of disease (β  =  0.25, *t* = 2.5, *P* = 0.014), age of onset (β  =  0.27, *t* = 2.7, *P* = 0.009), and borderline for LED (β  =  0.18, *t* = 1.9, *P* = 0.059). Confounder analysis for the LGN independent variable showed a significant model [*F*(4,102) = 11.6, *P* < 0.0001] with significant effects for LGN VAChT expression (standardized β = −0.32, *t* = −3.4, *P* = 0.001) and significant covariate effects for duration of disease (β  =  0.21, *t* = 2.0, *P* = 0.049), age of onset (β = 0.22, *t* = 2.2, *P* = 0.032), and for LED (β  =  0.26, *t* = 2.8, *P* = 0.0069). Confounder analysis for the entorhinal cortex independent variable showed a significant model [*F*(4,102) = 10.0, *P* = 0.0008] with significant effects for entorhinal cortex VAChT expression (standardized β = −0.23, *t* = −2.6, *P* = 0.011) and significant covariate effects for duration of disease (β  =  0.28, *t* = 2.7, *P* = 0.0085), age of onset (β  =  0.32, *t* = 3.3, *P* = 0.0015), and for LED (β  =  0.25, *t* = 2.4, *P* = 0.017).

The GPi (β =  0.15, *t* = 1.8, *P* = 0.074), lateral posterior nucleus thalamus (β = −0.11, *t* = −1.1, *P* = 0.26), caudate nucleus (β = −0.06, *t* = −0.7, *P* = 0.51) and anterior cingulum (β  =  0.01, *t* = 0.15, *P* = 0.88) did not survive confounder analysis.

**Figure 3:**
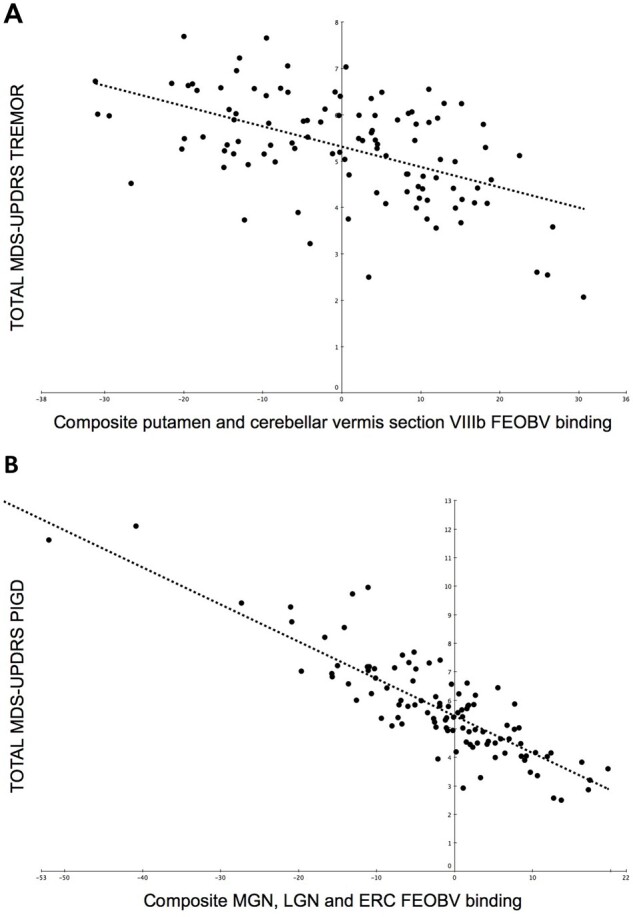
**Scatter plots of topographic FEOBV binding and clinical ratings for tremor scores ** (A** and PIGD ratings (B).** Motor ratings were adjusted for covariates of age of onset, duration of motor disease and levodopa equivalent dose. For the PIGD plot composite medial geniculate nucleus (MGN), lateral geniculate nucleus (LGN) and entorhinal cortex (ERC) FEOBV binding were used. The composite FEOBV binding was computed as the average percentage of mean binding (derived from the complete volumes of interest) in normal control persons for the three volumes of interest. Although the covariates may be driving some of the regressor effects the FEOBV binding achieved significance independent of the covariates (*F* = 28.16, *P* < 0.0001). For the tremor plot composite putamen and cerebellar vermis section of lobule FEOBV binding was also computed as the average percentage of mean binding in normal control persons and also achieved significance independent of the covariates (*F* = 4.51, *P* = 0.036).

A scatter plot of the regression between a composite FEOBV binding measure of the three major VOIs (MGN, LGN and entorhinal cortex) and total PIGD motor scores is shown in Fig. 3B. The composite FEOBV binding was computed as the average percentage of mean binding in normal control persons for the three VOIs because absolute binding averages could not be computed due to differences in binding intensity between these cortical and subcortical regions. The composite binding variable was significant in the model independent of the covariates (*F* = 28.16, *P* < 0.0001).

### Sub-analysis of non-episodic PIGD motor features

The total PIGD score is sum of episodic (i.e. freezing of gait and fall-related items) and non-episodic disturbances of impaired gait and balance ratings. We performed a sub-analysis using non-episodic PIGD motor scores to assess whether previously identified regions, such as the LGN, associating with the episodic mobility disturbances (gait freezing and falls) may also associate with non-episodic PIGD motor ratings (defined as the total PIGD motor score minus the ratings for items related to gait freezing and falls). Multiple regression with forward selection identified 3 VOIs where cholinergic terminal deficits correlated with non-episodic PIGD ratings: MGN (*F* = 25.93, *P* < 0.0001), entorhinal cortex (*F* = 8.86, *P* = −0.0038) and the GPi (*F* = 6.36, *P* = 0.014).

*Post hoc* analysis was performed to assess for possible confounder effects of age of onset, duration of disease and LED in multiple regression models. Analysis for the MGN independent variable showed a significant model [*F*(4,98) = 8.3, *P* < 0.0001] with significant effects for MGN VAChT expression (standardized β = −0.25, *t* = −2.6, *P* = 0.0097) and significant covariate effects for duration of disease (β  =  0.24, *t* = 2.3, *P* = 0.026), age of onset (β  =  0.30, *t* = 2.8, *P* = 0.0055), and non-significant trend for LED (β  =  0.16, *t* = 1.6, *P* = 0.11). Analysis for the entorhinal cortex independent variable showed a significant model [*F*(4,102) = 8.6, *P* < 0.0001] with significant effects for entorhinal cortex VAChT expression (standardized β = −0.23, *t* = −2.6, *P* = 0.011) and significant covariate effects for duration of disease (β  = 0.25, *t* = 2.4, *P* = 0.02), age of onset (β  =  0.32, *t* = 3.1, *P* = 0.0024) and LED (β  =  0.20, *t* = 2.1, *P* = 0.041). Analysis for the GPi independent variable showed a significant model [*F*(4,102) = 8.1, *P* < 0.0001] with significant effects for GPi VAChT expression (standardized β  =  0.20, *t* = 2.3, *P* = 0.025) and significant covariate effects for duration of disease (β  =  0.29, *t* = 2.7, *P* = 0.0075), age of onset (β  =  0.40, *t* = 4.2, *P* < 0.0001) and LED (β  =  0.25, *t* = 2.5, *P* = 0.013).

### PET-clinical correlations of the model-selected variables and post hoc analysis: tremor domain

Multiple regression with forward selection identified six VOIs where cholinergic terminal changes correlated with tremor ratings (Table 2). *Post hoc* analysis was performed to assess for possible confounder effects of age of onset, duration of disease and LED in multiple regression models.

Analysis for the putamen independent variable showed a significant model [*F*(4,102) = 2.5, *P* = 0.046] with significant effects for putamen VAChT expression (standardized β = −0.23, *t* = −2.3, *P* = 0.024) but no significant covariate effects for the confounder variables. Analysis for the cerebellar vermis section of lobule VIIIb independent variable showed a significant model [*F*(4,102) = 2.6, *P* = 0.041] with significant effects for cerebellar vermis section of lobule VIIIb VAChT expression (standardized β = −0.22, *t* = −2.4, *P* = 0.021) but no significant covariate effects for the confounder variables. A sample plot of the relationship between putaminal and cerebellar vermis section of lobule VIIIb FEOBV binding and tremor scores is shown in Fig.  3A. The composite FEOBV binding was computed as the average percentage of mean binding in normal control persons for the two VOIs and was significant independent of the covariates (*F* = 4.51, *P* = 0.036).

### Sub-analysis of resting and postural-kinetic tremor sub-scores

We performed a sub-analysis using resting and postural-kinetic tremor sub-scores because of possible pathophysiological differences between these two types of tremor.

For the resting tremor sub-scores, multiple regression with forward selection identified a single VOI cerebellar vermis section of lobule VIIIa (*F* = 6.4, *P* = 0.013). *Post hoc* analysis was performed to assess for possible confounder effects of age of onset, duration of disease and LED in multiple regression models. Analysis for the cerebellar vermis section of lobule VIIIa independent variable showed a non-significant but trending model [*F*(4,102) = 2.1, *P* = 0.083] with significant effects for cerebellar vermis section of lobule VIIIa VAChT expression (standardized β = −0.22, *t* = −2.3, *P* = 0.027) without significant covariate effects for the confounder variables.

For the postural-kinetic tremor sub-scores multiple regression with forward selection identified two VOIs: paracentral cortex (*F* = 8.5, *P* = 0.0046) and putamen (*F* = 5.1, *P* = 0.027). *Post hoc* analysis was performed to assess for possible confounder effects of age of onset, duration of disease and LED in multiple regression models. Analysis for the paracentral cortex independent variable showed a non-significant model [*F*(4,102) = 2.9, *P* = 0.025] with significant effects for paracentral cortex VAChT expression (standardized β = −0.21, *t* = −2.1, *P* = 0.042) without significant covariate effects for the confounder variables. Analysis for the putamen independent variable showed a non-significant model [*F*(4,102) = 2.6, *P* = 0.04] with a non-significant trending effect for putamen VAChT expression (standardized β = −0.17, *t* = −1.7, *P* = 0.084) without significant covariate effects for the confounder variables.

### PET-clinical correlations of the model-selected variables and post hoc analysis: rigidity domain

Multiple regression with forward selection identified 3 VOIs where cholinergic terminal changes correlated with rigidity ratings (Table 2). *Post hoc* analysis was performed to assess for possible confounder effects of age of onset, duration of disease and LED in multiple regression models. None remained significant in *post hoc* confounder analyses.

### PET-clinical correlations of the model-selected variables and post hoc analysis: distal limb bradykinesia domain

Multiple regression with forward selection identified 4 VOIs where cholinergic terminal changes correlated with bradykinesia ratings (Table 2). *Post hoc* analysis was performed to assess for possible confounder effects of age of onset, duration of disease and LED in multiple regression models. Analysis for the GPe independent variable showed a significant model [*F*(4,103) = 4.9, *P* = 0.0011] with significant effects for GPe VAChT expression (standardized β  =  0.36, *t* = 3.9, *P* = 0.0097) and significant covariate effects for duration of disease (β  =  0.24, *t* = 2.3, *P* = 0.0002) without significant covariate effects of the confounder variables. Analysis for the paracentral cortex independent variable showed a significant model [*F*(4,103) = 2.6, *P* = 0.038] with significant effects for paracentral cortex VAChT expression (standardized β  =  0.26, *t* = 2.5, *P* = 0.013) without significant covariate effects of the confounder variables. The interposed nucleus (cerebellum) and the pulvinar anterior nucleus did not survive confounder analysis.

### Complementary whole brain voxel-based correlation analysis: PIGD motor domain

Supplemental whole-brain voxel-based PET-motor correlation analysis was performed as an independent analysis to assess for internal consistency between findings from the data-driven VOI selection analysis and the supplemental whole-brain voxel-based correlation. In addition, the supplemental voxel-based correlation allowed assessment for possible hemispheric asymmetries in binding correlations and assessment of possible smaller sized clusters of binding correlation within the larger-sized cortical VOIs.

**Figure 4:**
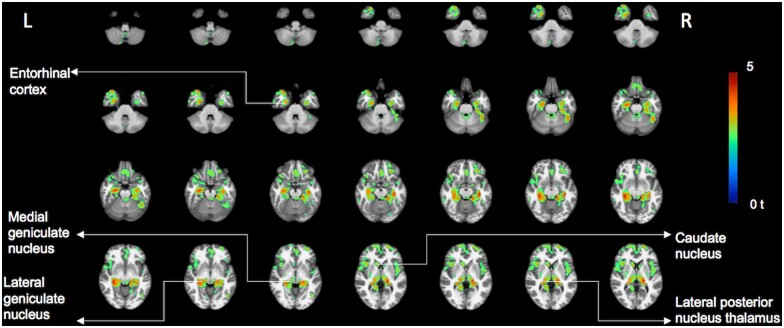
**Voxel-based vesicular acetylcholine transporter PET and total PIGD motor analysis**. Significant clusters of the inverse correlation between total PIGD motor scores and regional VAChT PET binding as shown in *t*-scores superimposed on brain MRI atlas images. (false discovery rate-corrected *P* < 0.05). The most prominent findings localized to the bilateral metathalamus (medial and geniculate nuclei), bilateral proximal optic radiations, bilateral thalamus proper (esp. the lateral posterior nucleus of the thalamus), bilateral fimbriae, right more than left mesiotemporal lobes (including entorhinal cortex and hippocampus), bilateral caudate nuclei, cingulum, especially anterior and mid portions, right operculum and bilateral prefrontal, pericentral and insular cortices. Additional smaller foci are seen in the cerebellum, including the right superior and posterior vermis. PET imaging findings superimposed on International Consortium for Brain Mapping (ICBM) adopted Montreal Neurological Institute (MNI) MRI T_1_-weighted template.

Whole brain voxel-based analysis showed regionally significant false discovery rate (FDR)-corrected (*P* < 0.05) correlations between the total PIGD motor scores and VAChT expression in the bilateral metathalamus (MGN and LGN), bilateral proximal optic radiations, bilateral thalamus proper (including the pulvinar and lateral posterior nucleus), bilateral fimbriae, right more than left mesiotemporal lobes (including entorhinal cortex and hippocampus), bilateral caudate nuclei, (anterior and mid) cingulum, right operculum, and bilateral prefrontal and insular cortices (Fig. 4). Additional correlations were seen in the pericentral cortices.

### Complementary voxel-based correlation analysis: tremor domain

**Figure 5:**
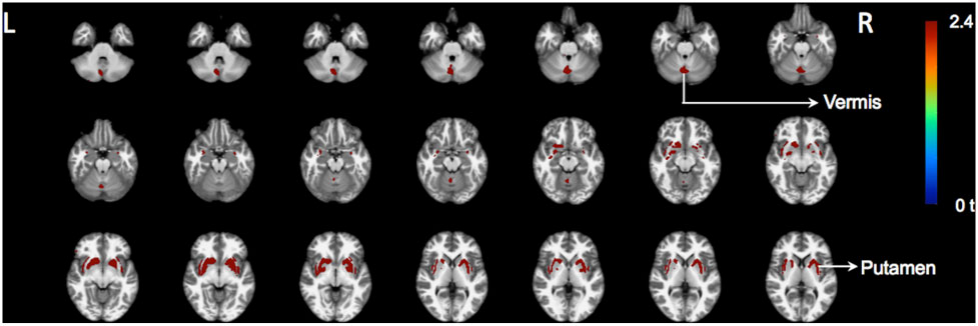
**Exploratory voxel-based vesicular acetylcholine transporter PET and total tremor motor correlation analysis**. Results of the exploratory voxel-based correlation analysis between cholinergic PET and tremor scores as shown in *t*-scores superimposed on brain MRI atlas images. Clusters of the inverse correlation between tremor motor scores and regional cholinergic PET binding are depicted (non-corrected at *P* = 0.01). Results of the explorative analysis shows regional binding in the bilateral putamen, anteroventral striatum, globus pallidus part externa, bilateral claustrum, and right superior and posterior vermis associating with tremor scores. PET imaging findings superimposed on International Consortium for Brain Mapping (ICBM) adopted Montreal Neurological Institute (MNI) MRI T_1_-weighted template.

An exploratory analysis (uncorrected at *P* = 0.01) was performed as no voxels survived FDR correction. The whole brain voxel-based exploratory analysis showed regionally correlations between the total tremor motor scores and VAChT binding in the bilateral putamen, anteroventral striatum, GPe, bilateral claustrum and right superior and posterior cerebellar vermis (Fig. 5). Additional correlations were seen in the cingulum.

### Complementary voxel-based correlation analysis: distal limb bradykinesia domain

**Figure 6:**
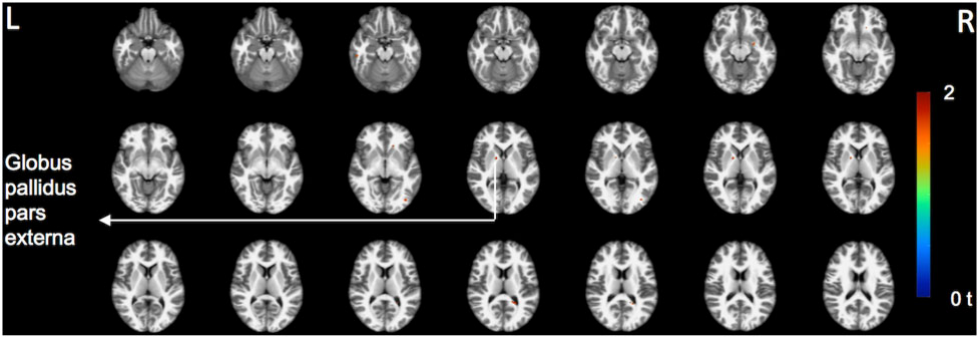
**Exploratory voxel-based vesicular acetylcholine transporter PET and distal limb bradykinesia motor correlation analysis**. Results of the exploratory voxel-based correlation analysis between cholinergic PET and distal limb bradykinesia scores as shown in *t*-scores superimposed on brain MRI atlas images. A small cluster of the positive correlation between distal limb bradykinesia scores and globus pallidus pars externa binding is depicted (non-corrected at *P* = 0.05). PET imaging findings superimposed on International Consortium for Brain Mapping (ICBM) adopted Montreal Neurological Institute (MNI) MRI T_1_-weighted template.

An exploratory analysis (uncorrected at *P* = 0.05) was performed as no voxels survived FDR correction. The whole brain voxel-based exploratory analysis showed positive correlations between total distal limb bradykinesia motor scores and VAChT binding in the GPe (Fig. 6). Negative correlations (not shown) included paracentral and mesiofrontal clusters in exploratory uncorrected analysis.

## Discussion

Cardinal motor features of Parkinson's disease are likely best explained by dysfunction of a basal ganglia–cortex–thalamus–cerebellum system rather than by isolated basal ganglia dysfunction.^11^ This system-level approach incorporates the concept that deficits within intersecting and overlapping component circuits underlie specific motor features of Parkinson's disease.^11^ The four major human brain cholinergic systems are the basal forebrain corticopetal (BF) projection system, cholinergic efferents of the peduncupontine-laterodorsal tegmental complex (PPN/LDTC), medial vestibular nucleus (MVN) cholinergic neurons projecting to the cerebellum, and striatal cholinergic interneurons (SChI). All three major cholinergic neurons-projection systems are important components of the larger basal ganglia–thalamus–cerebellar system, and clinical pharmacology suggests that alterations of cholinergic systems contribute to some cardinal motor features of Parkinson's disease.

Our prior acetylcholinesterase (AChase) PET imaging studies showed evidence of dissociable and additive mobility control deficits associated with BF (gait slowing and freezing) and PPN/LDTC (postural and sensory processing dysfunction) cholinergic terminal deficits.^28–30^ These studies were technically limited because AChase PET methods cannot reliably estimate tracer retention in regions with high tracer hydrolysis, including the striatum and cerebellar cortex.^31^ Unlike AChE PET, VAChT PET with [^18^F]-FEOBV has better anatomic resolution and also allows estimates of VAChT expression in both lower and high cholinergic terminal density brain regions, and more faithfully reproduces the regional distribution of cholinergic terminals described in post-mortem studies of human brain.^18^^,^^24^ Based on a large dataset of [^18^F]-FEOBV PET studies in Parkinson's disease subjects, we used a data-driven unbiased VOI-based selection data reduction method to identify brain regions where changes in cholinergic terminal density associated with cardinal motor impairments in Parkinson's disease. Supplemental voxel-based analyses were used to explore the possibility of expanded neural network changes associated with some cardinal motor features of Parkinson's disease.

A group comparison between the Parkinson’s disease subjects and normal older control persons showed evidence of widespread cortical (especially in the posterior cortices), and subcortical (especially thalamic and pallidal regions) but not in striatal or limbic regions in the patients. These findings indicate that cholinergic system changes in Parkinson’s disease have a prominent disease-specific component and cannot be fully explained by the effects of ageing.

PIGD motor features have limited to no responsiveness to dopamine replacement therapy in advancing Parkinson's disease, implicating changes in non-dopaminergic pathways in PIGD.^32^ Our prior AChase PET studies explicitly linked some regional cholinergic terminal deficits to gait dysfunction in Parkinson's disease.^28–30^ Our more recent cross-sectional studies with [^18^F]FEOBV PET suggested that Parkinson's disease subjects with episodic PIGD features (i.e. isolated falls and those with gait freezing) share common cholinergic terminal deficits in thalamus (LGN) and striatum (caudate nucleus). A visual attentional processing function of the LGN is consistent with a specific role of this region in episodic mobility disturbances. More extensive striatal, limbic, and prefrontal VAChT reductions were found in subjects with gait freezing.^10^

Our data-driven VOI selection approach identified specific cholinergic terminal deficits in several key brain regions associating with total PIGD motor scores, including episodic and non-episodic PIGD features: MGN, LGN, and entorhinal cortex. Our prior VAChT PET study identified the thalamus, esp. the LGN, and caudate nucleus as the most robust regional cholinergic deficit correlates of the episodic PIGD phenomena falls and gait freezing, respectively, in Parkinson's disease.^10^ Emerging evidence emphasizes an active role of the LGN in coordinating cortical regions for attending to relevant visual stimuli and tasks.^33^ A visual attentional processing function of the LGN is consistent with a specific role of this region in episodic mobility disturbances. Our prior VAChT PET study did not employ detailed anatomic VOI parcellation of the thalamic complex. Our current VOI-based selection results not only confirm a key role of the LGN but also of the MGN and entorhinal cortex in PIGD motor features. Our sub-analysis limiting PIGD ratings to non-episodic features identified the MGN, entorhinal cortex and GPi cholinergic terminal deficits but not the LGN cholinergic deficits as key correlates of non-episodic PIGD features. While conventionally conceived as an auditory system relay station, the MGN processes both auditory and non-auditory inputs, including vestibular, somatosensory, visual, and nociceptive inputs.^34^ In addition to corticopetal projections, efferent projections from the MGN include the caudate, putamen, amygdala, the ventromedial nucleus of the hypothalamus and the subparafascicular nucleus of the thalamus.^35^ These MGN efferents may constitute a pathway by which environment events can influence, without direct cortical participation, processes integrated in the striatum and limbic system.^35^

Effective postural control depends on successful integration of vestibular, somatosensory and visual information. The multisensory processing role of the MGN suggests that MGN cholinergic terminal deficits, likely reflecting degeneration of PPN/LDTC cholinergic afferents, contribute to impaired sensorimotor integration and non-episodic PIGD motor features in Parkinson's disease. Within the thalamus, the LGN and pulvinar play major roles in visual processing. There are, however, multiple projections from the vestibular nuclei to the thalamus. Primary thalamic targets include the MGN, LGN, ventrobasal, ventrolateral and intralaminar nuclei.^36^ These nuclei contain multisensory neurons that process and relay vestibular, proprioceptive and visual signals to the vestibular cortex. In non-human primates, the parieto-insular vestibular cortex has been proposed as the core vestibular region.^37^ Human studies show not only evidence of projections to the parieto-insular vestibular cortex but also to the somatosensory cortex, medial superior temporal sulcus area, intraparietal sulcus and hippocampus that may explain the large influence of vestibular signals on self-motion perception, spatial navigation, internal models of gravity, one's body perception and bodily self-consciousness via multimodal sensory processing.^37^ Injections in the caudate, putamen and amygdala retrogradely labelled the medial division of the MGN.

Our prior voxel-based FEOBV PET analysis showed evidence of diffuse limbic cholinergic dysfunction (hippocampus, entorhinal cortex, amygdala, and anterior cingulate cortex) in Parkinson's disease subjects with gait freezing.^10^ Our current findings suggest a preferential role of entorhinal cortical cholinergic deficits in non-episodic PIGD motor features. The entorhinal cortex is the major input and output structure of the hippocampal formation and BF cholinergic afferents are key modulators of entorhinal cortex and hippocampal functions. Entorhinal cortices transmit multimodal sensory information to the hippocampus and contain a directionally oriented, topographically organized neural map of the spatial environment.^38–40^ The entorhinal cortex plays a role in multimodal information processing relevant for spatial navigation, including multiple head direction signals,^41^ which arise from inputs from the vestibular system and then ascend from brainstem regions through thalamic nuclei, including the MGN and LGN.^36^ Loss of vestibular input has shown to result in hippocampal atrophy in patients with bilateral vestibular failure and deficits in spatial memory suggesting that spatial navigation critically depends on preserved vestibular function.^42^ A MRI study found significant structural volume correlations in the hippocampus, entorhinal cortex, and thalamus that related to first person navigational accuracy.^43^ These observations support the theory that the entorhinal cortex, hippocampus and thalamus are key structures for updating position and orientation during ground-level navigation.^43^ Impaired integrity of the entorhinal cortex may directly affect ambulatory functions that depend on safe navigation of the environment.

It is well known from models of the basal ganglia that the striatal nuclei provide inhibitory control over the GPi and substantia nigra pars reticulata,^44^ effectively releasing the tonic GABA-ergic inhibition mediated by the output structures of the basal ganglia. As such, any hypoactivity within the striatum would therefore lead to a relative increase in inhibitory outflow from the basal ganglia.^45^ We found evidence of a positive correlation between the cholinergic nerve terminal integrity of the GPi and severity of non-episodic PIGD motor features. This may potentially suggest relative upregulation of cholinergic nerve terminals in the GPi but will need to be confirmed in future studies. The association between non-episodic PIGD ratings and GPi cholinergic deficits is intriguing. Altered GPi function induced by bilateral deep brain stimulation (DBS) is associated with worsening postural stability and gait dysfunction. This is well established in PD, and is also documented in patients undergoing deep brain stimulation for cervical dystonia without prior prominent gait disorders.^46^ Our results suggest cholinergic PPN-LDT afferents play an important role in this aspect of GPi function.

The confounder analysis also showed significant independent duration of disease effect and ageing effects implying that cholinergic dysfunction in the MGN, LGN and entorhinal cortex represent a later stage disease phenomenon in keeping with the natural history of disease progression in Parkinson's disease.

A supplemental whole-brain-based analysis provided independent validation for the model-selected VOI analysis showing showed significant clusters of an inverse correlation between total PIGD motor scores and regional VAChT PET binding. The most prominent findings localized to the bilateral metathalamus (LGN and MGN), and the entorhinal cortex. Significant but less prominent findings included the bilateral proximal optic radiations, bilateral thalamus proper, bilateral fimbriae, right more than left mesiotemporal lobes (including entorhinal cortex and hippocampus), bilateral caudate nuclei, cingulum, especially anterior and mid portions, right operculum and bilateral prefrontal, pericentral and insular cortices. Additional smaller foci were seen in the cerebellum, including the right superior and posterior vermis. Among these regions the MGN, LGN and entorhinal cortex had most robust significance providing independent validation for the major findings of the model-selected VOI analysis. These findings support a system-network conception of the pathophysiology of PIGD motor features of Parkinson's disease.

Dysfunctional rhythms related to parkinsonian tremor may emerge within loops interconnecting the basal ganglia, cerebellum, thalamus, and cerebral cortex.^47^ Parkinsonian tremor may result from the combined dysfunctions within two circuits; basal ganglia abnormalities triggering tremor episodes and cerebello–thalamo–cortical circuit dysfunction that sustains tremor.^48^ GPi, GPe and putamen neurons are transiently activated at the onset of tremor episodes with pallidal (but not striatal) dopamine depletion correlated with clinical tremor severity.^49^ Clinical benefit of anti-muscarinic cholinergic drugs suggests a role for the cholinergic systems in tremor.^5^^,^^50^^,^^51^ Our initial model analysis identified cholinergic terminal deficits in putamen, GPe, some cortical, thalamic and cerebellar regions as correlates of Parkinson's disease tremor scores^52^ with the putamen and cerebellar vermis section of lobule VIIIb remaining significant in confounder variables. An supplemental exploratory whole-brain voxel-based analysis showed regional correlations between the tremor motor scores and VAChT binding in the bilateral putamen, anteroventral striatum, GPe, bilateral claustrum, right mid-cingulum and right superior and posterior cerebellar vermis. The findings would support a system-level of tremor pathophysiology in Parkinson's disease.

The clinical benefit of anti-muscarinic cholinergic drugs on symptoms of tremor in Parkinson's disease has been explained by a model of striatal imbalance between loss of dopamine and hypothesized upregulation of cholinergic neurotransmission, at least in patients with early-stage disease.^5^^,^^6^ Based on this longstanding ‘seesaw’ model, we anticipated finding positive rather than negative correlations between cholinergic binding levels and severity of the tremor. This may have several explanations. First, insofar a seesaw or striatal imbalance model exists it may be more at the cholinergic receptor level, including post-synaptic receptors. This may be compared to the PD-specific presynaptic nigrostriatal dopamine nerve terminal losses that at least in early-stage disease are associated with increased expression of post-synaptic DA receptors.^53^ If this may be true, then a presynaptic marker of the integrity of cholinergic nerve terminals may not be a good cholinergic marker to test this hypothesized model. Second, principal component analysis of whole brain cholinergic imaging studies has shown bidirectional changes where some regions of the brain may be relatively increased versus other regions relatively decreased.^54^ This may mean that presumed cholinergic correlations could be either negative or positive depending on the cholinergic expression state of a specific region of interest. Our findings of both negative and positive correlations of different regions in the distal limb bradykinesia analysis may be consistent with this. Third, our analysis was cross-sectional in nature across a wide range of disease stages and severities but did not include longitudinal assessment of participants in very early or de novo stage of disease to more properly test the seesaw model. Lastly, tremor-related brain changes are not limited to dopaminergic or cholinergic nerve terminal changes but may also involve noradrenergic or serotoninergic changes.^55^^,^^56^

Resting tremor is the most frequent tremor type and present in the majority of the Parkinson's disease patients. However, action tremor is also present in nearly one-third of Parkinson's disease patients.^57^ Our sub-analysis of resting versus postural-kinetic tremor sub-scores demonstrated regional differences associating with specific cholinergic brain regions. Resting tremor tended to associate with vermis section of lobule VIIIa whereas postural-kinetic tremor scores associated with the paracentral lobule and borderline with the putamen. This is likely explained by differences in pathophysiology of these different manifestations of tremor in Parkinson's disease.^55^

The pathophysiology of rigidity remains largely unknown. Prior studies showed abnormalities in long-latency stretch reflexes and stretch-induced co-activation of agonist-antagonistic muscles.^58–60^ Mechanisms involved in postural muscle tone regulation and in locomotor rhythm generation are integrated so that the appropriate locomotor movements can be achieved.^61^ Cholinergic neurons of the mesopontine tegmentum may simultaneously regulate the level of muscle tone and locomotor rhythm by modulating the activities of the lower brainstem reticulospinal neurons and central pattern generators in the spinal cord.^61^ These are not regions easily amenable to quantification of cholinergic terminal density with PET.

Our initial analysis showed that occipital cortex cholinergic terminal density of the occipital cortex and the cerebellar hemispheric section of lobule V had inverse correlations with rigidity scores. Neither the cortical or cerebellar regions remained significant in post hoc confounder analyses. Cortical cholinergic losses first manifest in the occipital cortex in early Parkinson's disease^62^ and model selection of the occipital cortex may reflect severity of cholinergic cortical losses rather than an intrinsic occipital cortical role of rigidity. There is increasing recognition that the cerebellum plays a role in cardinal motor features other than tremor.^63^^,^^64^ Our results suggest that presynaptic cholinergic nerve terminal integrity of these regions is not a major driver of rigidity in Parkinson's disease but does not exclude more relevant roles of cholinergic signalling in more caudal CNS regions.

Distal limb bradykinesia is the disease-defining feature of Parkinson's disease.^65^ Bradykinesia is conventionally considered the consequence of a failure of basal ganglia output to the primary motor cortex.^66^ Bradykinesia is the cardinal motor symptom most closely related to the reduction in striatal dopamine^67^ with a possible smaller role of thalamic dopaminergic nerve terminals.^68^ Striatal dopaminergic neurotransmission is a critical modulator of what is termed vigour—the speed, strength, or amplitude of actions.^69–71^ Bradykinesia is best interpreted as diminished vigour secondary to striatal dopaminergic deficits driving network dysfunction that includes the basal ganglia and interconnected structures, such as the primary motor cortex.^72^ Consistent with a major role for striatal dopamine deficiency driving bradykinesia, levodopa therapy, but not anti-cholinergic treatment, significantly improves bradykinesia, but DRT does not restore normal function and many aspects of DRT effects remain poorly understood.^69^^,^^72^ SChIs modulate striatal and basal ganglia circuitry based on a interplay of dopaminergic and cholinergic signalling.^73^ Recent studies suggest that SChIs play an important role in action selection, as opposed to movement vigour,^74^ findings consistent with little anti-muscarinic cholinergic treatment effects on bradykinesia. Our analysis demonstrated significant VAChT PET-motor correlation coefficients in 2 regions: GPe and the paracentral cortex. This may suggest differential expression of cholinergic nerve terminals in regions relevant for distal limb bradykinesia. The association between GPe cholinergic deficits and bradykinesia ratings is consistent with a large body of data indicating an important integrative role of GPe neurons in basal ganglia function.^75^ The GPe appears to be a hub coordinating the activity of other basal ganglia nuclei and is likely to be a key node regulating movement vigour and action selection. Animal model experiments indicate that GPe neuron dysfunction is associated with generation of the 12–30 Hz (beta range) basal ganglia oscillations strongly associated with bradykinesia.^76^ Optogenetic excitation of GPe neurons improves bradykinesia secondary to dopamine depletion in a murine model of parkinsonism.^77^ The positive effects of optogenetic excitation was seen with stimulation of the parvalbumin-containing subpopulation of GPe neurons. Our results suggest that normal GPe cholinergic innervation may be particularly important for the normal function of this subpopulation of GPe neurons. Positive correlations between GPe VAChT binding and clinical ratings may possibly related to observations that striatal indirect and subthalamic nucleus input organizations present cell-type-specific integration in the GPe leading to opposite activity patterns between prototypic and arkypallidal neurons and opposite behavioural outcomes on movement inhibition during locomotor activity.^78^ The association between cholinergic nerve terminals in the paracentral cortex and distal limb bradykinesia may implicate the importance of the primary motor cortex with a framework of Parkinson's disease as a system-level disorder with interactions between different components of the entire basal ganglia–cortex–thalamus–cerebellum system rather than from isolated basal ganglia dysfunction.^11^ The distal limb bradykinesia findings of the GPe and paracentral cortex are preliminary in nature and need independent verification in future studies given the absence of robust independent confirmation in whole brain voxel-based correlation analysis.

A limitation of the study is the male predominance of our patient population limiting generalization of our findings to a larger female Parkinson's disease population. Our cholinergic system assessment consisted of a single presynaptic marker and did not include any measures of post-synaptic functions, such as cholinergic receptors that might cast light on more complex interactions. Although *post hoc* confounder analysis did not indicate independent correlates of regional cholinergic system changes for rigidity, a more dynamic role of cholinergic systems interacting with other systems is likely. Our *post hoc* confounder analysis was limited by a smaller subset of participants who completed DTBZ scans. However, demographic and clinical disease variables were similar between the different subsets of patients in this study. Another limitation of DTBZ PET is that it does not allow dopaminergic nerve terminal assessment outside the striatum. Finally, some smaller cerebellar and thalamic parcellations could not be sampled by PET.

Our results are consistent with Parkinson's disease clinical pharmacology, key clinical observations, and present neurobiological understanding of these cholinergic systems. Our association of metathalamus (MGN and LGN) and entorhinal cortex cholinergic deficits with PIGD features is consistent with the likely integrative roles of metathalamus structures in multi-modal sensory processing and spatial navigation, respectively. Similarly, our results are consistent with the deleterious effects of anti-cholinergic agents on PIGD features of Parkinson's disease. The medial and lateral geniculate nuclei, and entorhinal cortex likely play a pivotal yet underexamined role in balance and gait functions in Parkinson's disease. Independent covariate effects of (longer) duration of disease and older age suggest that cholinergic effects on PIGD motor features represent a combination of ageing and Parkinson's disease-specific effects. Our association of putaminal and cerebellar cholinergic deficits with tremor ratings is consistent with the basal ganglia representing a key node within loops interconnecting the basal ganglia, cerebellum, thalamus and the cerebral cortex in tremor pathophysiology. Our results also suggest significant and distinct consequences of degeneration of cholinergic PPN-LDT afferents to both segments of the globus pallidus. These observations suggest that targeting cholinergic neurotransmission in the GPi versus GPe may a plausible avenue to treat important aspects of motor dysfunction in PD.

In conclusion, among the principal motor features of Parkinson's disease, our data-driven approach identified key brain regions where cholinergic deficits are associated with PIGD motor features, distal limb bradykinesia and tremor.

Non-specific regional cholinergic terminal deficit associations with rigidity likely reflect more complex multifactorial signalling mechanisms with smaller contributions from cholinergic pathways. Emphasizing the utility of a systems-network conception of the pathophysiology of Parkinson's disease cardinal motor features, our results are consistent with specific deficits in BF, PPN/LDTC and MVN pathways, against the background of nigrostriatal dopaminergic deficits, contributing significantly to the cardinal motor features of Parkinson's disease.

## Supplementary material

Supplementary material is available at *Brain Communications* online.

## Supplementary Material

fcab109_Supplementary_Data

## Abbreviations

AChase =: acetylcholinesterase
BF =: basal forebrain corticopetal
DTBZ =: [^11^C]-dihydrotetrabenazine
FEOBV =: [^18^F]-fluoroethoxybenzovesamicol
Gpe =: globus pallidus pars externa
Gpi =: globus pallidus pars interna
LGN =: lateral geniculate nucleus
MVN =: medial vestibular nucleus
MGN =: medial geniculate nucleus
PIGD =: postural instability and gait difficulties
PPN/LDTC =: pedunculopontine nucleus/lateral dorsal tegmental complex
SchI =: striatal cholinergic interneurons
VAChT =: vesicular acetylcholine transporter
VOI =: volumes of interest
