# Supplementary material for: Regional cerebral cholinergic nerve terminal integrity and cardinal motor features in Parkinson’s disease
Source: Brain Commun. 2021 May 22;3(2):fcab109. doi: 10.1093/braincomms/fcab109 (PMC8196256; doi:10.1093/braincomms/fcab109)
Supplement: fcab109_Supplementary_Data [file fcab109_supplementary_data.pdf]

Supplemental figure. Normal cerebral biodistribution pattern of FEOBV PET. Most intense uptake is present in the basal ganglia, followed by the thalamus, vermis, hippocampus and lowest in the neocortex.

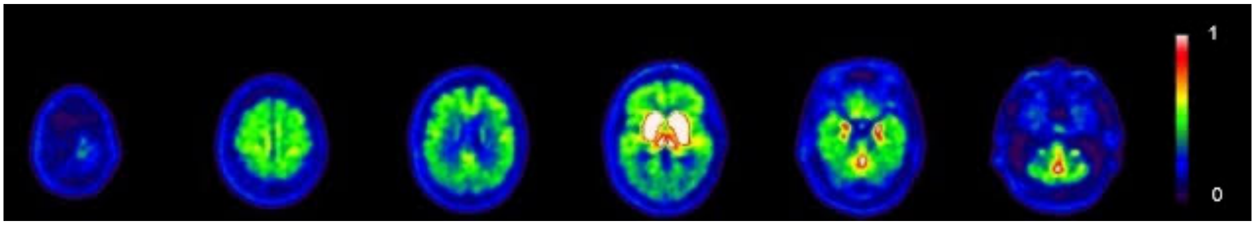

Supplemental Table. Listing of FEOBV PET volumes of interests (VOIs) and respective volumes (mm^3). Hemispheric VOIs are bilaterally averaged.

**VOI**	**Volume mm^3**
Freesurfer atlas	
Anterior cingulum	6434
Accumbens	2033
Amygdala	3902
Caudate	10227
Frontal lobe	147405
Hippocampus	11657
Occipital lobe	79972
Parietal lobe	75836
Posterior cingulum	10771
Putamen	16125
Temporal lobe	88303
Entorhinal cortex	4539
Fusiform gyrus	21983
Parahippocampal gyrus	3270
Paracentral cortex	8071
Postcentral cortex	18271
Precentral cortex	26848
Insula	13893
Globus pallidus pars interna	1303
Globus pallidus pars externa	2869
Cerebellar atlas	
cerebellar hemisphere lobules I-IV	10386
cerebellar hemisphere lobule V	12020
cerebellar hemisphere lobule VI	23988
vermis section of lobule VI	2768
cerebellar hemisphere lobule VIIa crus I	35584
cerebellar hemisphere lobule VIIa crus II	25747
vermis section of crus II	587
cerebellar hemisphere lobule VIIb	13236
vermis section of lobule VIIb	259
cerebellar hemisphere lobule VIIIa	13116
vermis section of lobule VIIIa	1554
cerebellar hemisphere lobule VIIIb	11304
vermis section of lobule VIIIb	798

cerebellar hemisphere lobule IX	9538
vermis section of lobule IX	1019
cerebellar hemisphere lobule X	1835
vermis section of lobule X	465
dentate nuclei	4094
interposed nuclei	281
fastigial nuclei	108
Thalamic complex atlas	
lateral geniculate nucleus	426
medial geniculate nucleus	187
pulvinar lateral nucleus	446
pulvinar medial nucleus	2067
ventral lateral posterior nucleus	1647
centromedian nucleus	496
ventral lateral anterior nucleus	1176
pulvinar anterior nucleus	411
mediodorsal medial magnocellular nucleus	1341
parafascicular nucleus	120
mediodorsal lateral parvocellular nucleus	478
central medial nucleus	114
ventral anterior nucleus	710
pulvinar lateral nucleus	381
anteroventral nucleus	235
ventral lateral posterior nucleus	1569
lateral posterior nucleus	210
Globus pallidus VOIs	
Globus pallidus pars interna	1303
Globus pallidus pars externa	2869
